# Evaluation of Topology Optimization Using 3D Printing for Bioresorbable Fusion Cages: A Biomechanical Study in a Porcine Model

**DOI:** 10.1097/BRS.0000000000004491

**Published:** 2022-09-20

**Authors:** Nathan C. Ho, Scott J. Hollister, Virat Agrawal, Colleen L. Flanagan, Chloe Lee, Matthew B. Wheeler, Huan Wang, Edward Ebramzadeh, Sophia N. Sangiorgio

**Affiliations:** a The J. Vernon Luck, Sr., M.D. Orthopaedic Research Center, Orthopaedic Institute for Children and UCLA Department of Orthopaedic Surgery, Los Angeles, CA; b 3D Center for Medical Fabrication and Coulter Department of Biomedical Engineering, Georgia Institute of Technology, Atlanta, GA; c Department of Biomedical Engineering, The University of Michigan, Ann Arbor, MI; d Department of Bioengineering and Carl Woese Institute for Genomic Biology, University of Illinois, Urbana-Champaign, IL; e RE3 Innovative Neuroscience Institute, P.L.L.C, Sarasota, FL

**Keywords:** cervical cage, anterior cervical discectomy and fusion, bioresorbable, topology optimized, 3D printing, polycaprolactone, swine model

## Abstract

Preclinical biomechanical study of topology optimization versus standard ring design for bioresorbable poly-ε-caprolactone (PCL) cervical spine fusion cages delivering bone morphogenetic protein-2 (BMP-2) using a porcine model.

**Objective.:**

The aim was to evaluate range of motion (ROM) and bone fusion, as a function of topology optimization and BMP-2 delivery method.

**Summary of Background Data.:**

3D printing technology enables fabrication of topology-optimized cages using bioresorbable materials, offering several advantages including customization, and lower stiffness. Delivery of BMP-2 using topology optimization may enhance the quality of fusion.

**Methods.:**

Twenty-two 6-month-old pigs underwent anterior cervical discectomy fusion at one level using 3D printed PCL cages. Experimental groups (N=6 each) included: Group 1: ring design with surface adsorbed BMP-2, Group 2: topology-optimized rectangular design with surface adsorbed BMP-2, and Group 3: ring design with BMP-2 delivery via collagen sponge. Additional specimens, two of each design, were implanted without BMP-2, as controls. Complete cervical segments were harvested six months postoperatively. Nanocomputed tomography was performed to assess complete bony bridging. Pure moment biomechanical testing was conducted in all three planes, separately. Continuous 3D motions were recorded and analyzed.

**Results.:**

Three subjects suffered early surgical complications and were not evaluated. Overall, ROM for experimental specimens, regardless of design or BMP-2 delivery method, was comparable, with no clinically significant differences among groups. Among experimental specimens at the level of the fusion, ROM was <1.0° in flexion and extension, indicative of fusion, based on clinically applied criteria for fusion of <2 to 4°. Despite the measured biomechanical stability, using computed tomography evaluation, complete bony bridging was observed in 40% of the specimens in Group 1, 50% of Group 2, 100% of Group 3, and none of the control specimens.

**Conclusion.:**

A topology-optimized PCL cage with BMP-2 is capable of resulting in an intervertebral fusion, similar to a conventional ring-based design of the same bioresorbable material.

Anterior cervical discectomy followed by fusion is the standard treatment for end-stage cervical degenerative disk disease. Traditionally, autologous iliac bone graft has been used;^1^–^3^ however, complications such as infections, fractures, and donor site pain have led to the use of cages.^4^–^7^ While metallic cages have addressed complications related to bone graft, new issues, including cage migration, subsidence, and stress shielding have arisen.^8^–^11^ Alternative materials, such as carbon fiber and polyether-ether-ketone, have been introduced to address concerns with stress shielding.^8^–^10^,^12^–^15^ In addition, these materials are radiolucent, allowing enhanced postoperative imaging during healing.^11^,^16^–^18^ Despite these potential advantages, reports of subsidence and breakage persist.^19^,^20^

Bioresorbable materials such as polylactic acid (PLA), which have a comparable modulus of elasticity to bone, have also been explored;^21^ however, cracks and foreign body reactions have been reported.^22^,^23^ Poly-ε-caprolactone (PCL), another biocompatible and bioresorbable polymer with comparable mechanical properties, offers the additional potential advantage that it can be chemically modified to integrate with bone morphogenetic protein-2 (BMP-2).^24^ Further, PCL displays a slower degradation profile with fewer acidic byproducts than PLA, suggesting that it might be preferable as an implant choice.^24^–^26^

BMP-2 has been used in fusion procedures as a supplement to bone graft to enhance osteoinductive activity.^27^–^30^ BMP-2 is approved for lumbar fusion, but is often used off-label for anterior cervical discectomy followed by fusion. Despite these potential advantages, complications such as osteolysis, neck edema, swelling, airway compression, and dysphagia have been reported.^27^,^29^,^31^–^36^ It is hypothesized that these complications arise due to BMP-2-induced inflammatory response.^29^,^33^,^35^,^36^ In addition, BMP-2 is highly soluble; therefore, the molecules may migrate from the planned fusion site and diffuse into adjacent tissues and promote ectopic bony fusion. Currently, the FDA has approved the collagen sponge carrier to deliver BMP-2. An effective delivery method has the potential to lessen these complications if it can localize BMP-2 at the targeted site long enough to induce osteoinduction.^35^,^37^,^38^

Advances in 3D printing have allowed for fabrication of topology-optimized fusion cages, which have defined structural layouts and inner microstructures. Topology optimization is a mathematical method that spatially optimizes material distribution by providing optimum implant stiffness, material content, and porosity. Conventional shapes for cages are typically ring-shaped or boxed, intended to provide immediate strength to maintain disk height and shield bone graft material within the cage.^39^ Topology optimization in combination with laser sintering enables fabrication of complex morphologies using bioresorbable polymers, potentially addressing design constraints of conventional devices, maximizing stability, reducing stress shielding, and generating the desired porosity for biofactor delivery.^40^–^42^

The goal of the present study was to evaluate the effect of bioresorbable PCL cage design and BMP-2 delivery method on fusion in a large animal model in three experimental groups. The primary outcome variable was range of motion (ROM) evaluated by pure moment kinematic testing. In addition, bony bridging was assessed from nanocomputed tomography (CT) images.

## METHODS

**Figure 1:**
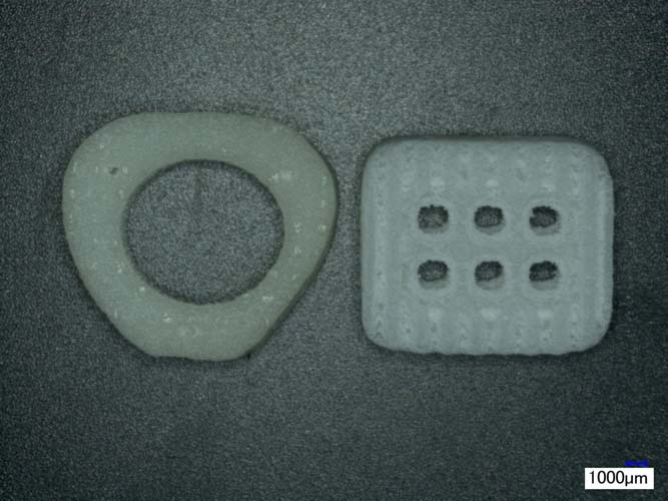
(Left) Ring design cage based on a commercially available device; (right) topology-optimized rectangular design.

Two biodegradable cervical cage designs composed of PCL were included: (1) a ring-shaped cage, designed based on commercially available fusion cages and (2) a rectangular-shaped design with topology optimization. The ring-shaped design consisted of a cylindrical cervical cage with a large central hole and small teeth on the endplate interfaces, to avoid expulsion. The rectangular design was created using a modular approach, combining topology optimization for the porous regions with image-based design for the cage shape and serrated fixation ridges (Fig. 1). Implants were fabricated using a laser sintering approach previously described.^40^,^41^

Two BMP-2 delivery methods were included: (1) chemically adsorbed and (2) collagen sponge. PCL can be chemically modified to integrate with BMP-2.^24^ Collagen sponge delivery could not be tested with the topology-optimized design as this shape does not allow for insertion of a sponge. BMP-2 was dissolved in 20 mM acetic acid (1 mg/mL) and then further diluted in conjugation buffer to give a total BMP-2 delivery 0.5 mg. BMP-2 was then adsorbed to cages by placing the cages in BMP-2 solution for 30 minutes in the operating room. For collagen sponge delivery, ACS sponges (Medtronic) were also placed in BMP-2 solution for 30 minutes in the operation room.

### Experimental Groups

Three experimental groups were included in the present study: Group 1: ring design with adsorbed BMP-2; Group 2: topology-optimized rectangular design with adsorbed BMP-2; Group 3: ring design with BMP-2 delivery via collagen sponge (total N=18). In addition specimens (N=4), two of each design, were included as controls with no BMP-2 or bone graft.

### Surgery

A total of 22 Yorkshire pigs were initially included. Surgeries were performed at ~100 kg at 6 months of age in approved facilities. All procedures were conducted under protocol (#11173) approved by the Institutional Animal Care and Use Committee (IACUC) at the University of Illinois. The surgical approach consisted of an anterior exposure of the cervical spine at the C3-C4, C4-C5, or C5-C6 level. The animal, after intubation and general anesthetic, was positioned dorsally on the operating table. Its neck was prepped and draped and a linear incision made from the midline sternal notch to the left mandible following the sternocleidomastoid line. The incision was made with a #10 scalpel and monopolar cautery to extend the incision through the platysma until a plane was developed between the sternoclediomastoid muscle and neurovascular bundle laterally and the trachea and esophagus medially. This plane was developed down to the anterior longitudinal ligament of the spinal column. Self-retaining retractors were placed in the wound with special attention not to harm the esophagus and/or neurovascular bundle. The disk was incised and removed with a pituitary type rongeur. An interbody distractor was placed in the disk space to permit insertion of the cage. After achieving meticulous hemostasis, the deep tissue layers were closed using vicryl suture.

### Specimen Preparation

Six months after fusion surgery, all pigs were sacrificed, and complete cervical spines were harvested. High-resolution digital radiographs were taken to document the condition of the harvested spines and to confirm the level of fusion. In addition, prior to mechanical testing all vertebrae were scanned on a GE Nanotom M Nano-CT system at a resolution of 30 microns/voxel, fine enough to show trabecular bone structure. Resulting images were viewed in Mimics (Materialise). All 3D image data were assessed in each plane through the whole volume to determine if complete bony bridging occurred across the fusion site.

Specimens were dissected to remove all muscular tissues but retain ligamentous structures. The cranial and caudal vertebrae of each specimen were potted using a low temperature curing resin. To ensure proper anatomic alignment with the axis of the load frame, and that no off-axis loads were applied, two triplanar laser levels were used.^43^

Motion tracker flags were placed using custom flag-holders on each of the most cranial and caudal vertebrae to capture the motion of the complete cervical spine segment. Two additional flag-holders were placed on the vertebrae at the level of fusion, to capture the motion of the functional spine unit (FSU) of interest. Radiographs were taken to confirm and document the proper placement of each flag holder prior to testing.

### Biomechanical Testing

**Figure 2:**
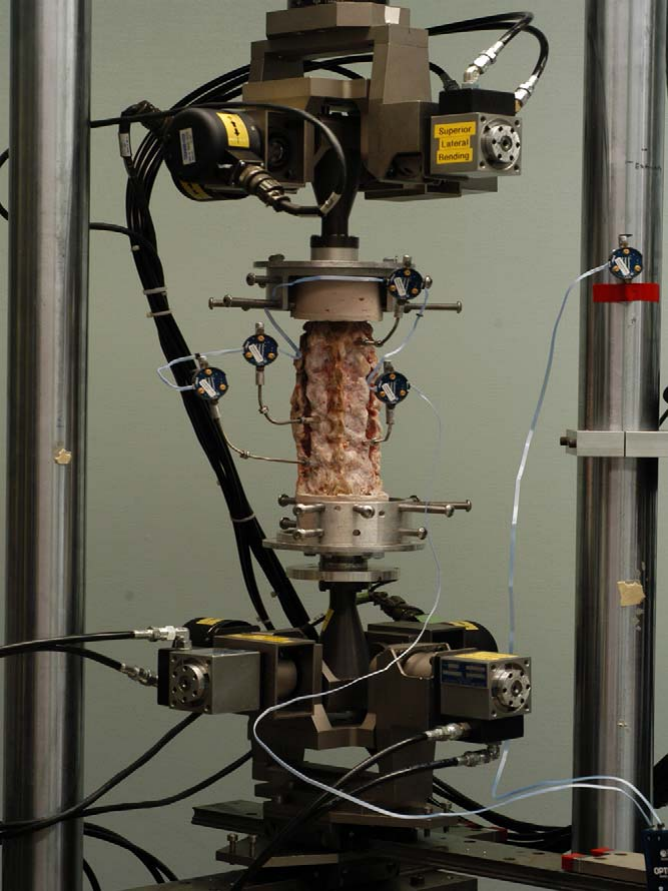
Biomechanical test setup in 8-degree-of-freedom (DOF) Mini Bionix 858 FlexTest System.

The biomechanical testing was conducted using an MTS 858 minibionix servohydraulic load frame equipped with the FlexTest System (MTS Systems) as described previously.^43^–^45^ Each specimen was mounted with the cranial end attached to the upper gimbals of the load frame and the caudal end attached to the lower gimbals. The lower gimbals were mounted on a custom table with two freely moving orthogonal linear bearings in the transverse plane, to minimize shear loading and enable pure moment testing. The specimens were rigidly attached to the load frame, such that the anatomical axes of the specimen were aligned with the axes of the load frame (Fig. 2).

Each spine was subjected to three separate pure bending moments in each of the following directions: axial rotation, lateral bending, and flexion/extension.^46^ The parameters for testing were based on previous *in vitro* cervical porcine studies.^47^–^49^ First, left and right axial torsion was applied to a peak of ±2.5 Nm. Next, left and right lateral bending was applied to a peak of ±2.0 Nm. Finally, flexion and extension was applied to a peak of ±2.0 Nm. For each of three bending profiles, all other degrees of freedom were unrestricted. In particular, axial load was continuously maintained at zero and motions of the caudal vertebrae in the horizontal plane were unrestricted using two low friction linear bearings. All three loading sequences were performed for six cycles to allow for two cycles of preconditioning and four cycles of data collection. This minimized the viscoelastic effects that may skew results in the beginning of the tests. No coupled moments were applied in the present study.

Motion of the complete cervical spine segment as well as the FSU of interest were recorded throughout testing using an Optotrak 3020 3D motion tracker system (Northern Digital Inc.). The motion tracker system has a reported accuracy of 0.1 mm and 0.1°, and a resolution of 0.01 mm.^50^ Each motion tracker flag was equipped with three noncollinear, light emitting diode markers. An additional flag was mounted on the load frame to create a local fixed coordinate axis.

### Statistical Analysis

Hysteresis curves for each plane and direction of rotation were analyzed to determine the ROM of the entire cervical spine and the functional spinal unit that was fused. Kruskal-Wallis nonparametric analysis was used to compare the experimental groups, followed by median tests, to compare each pair of experimental groups. Box plots were constructed to present the entire distribution of data for each experimental group. For the evaluation of outcome, flexion and extension of 1° or less was considered to be represent clinically successful fusion. This threshold was selected for consistency with previously defined clinical criteria.

## RESULTS

Three of the 18 animals in the experimental groups suffered complications during surgery and were excluded from the analysis, rendering a total of 15 harvested cervical spines in the experimental groups, and four control specimens. Specifically, the number of specimens available for testing were as follows: Group 1=5, Group 2=6, and Group 3=4. During data collection, the motion capture flag of two specimens malfunctioned. Therefore, two specimens were excluded from some analyses.

**Figure 3:**
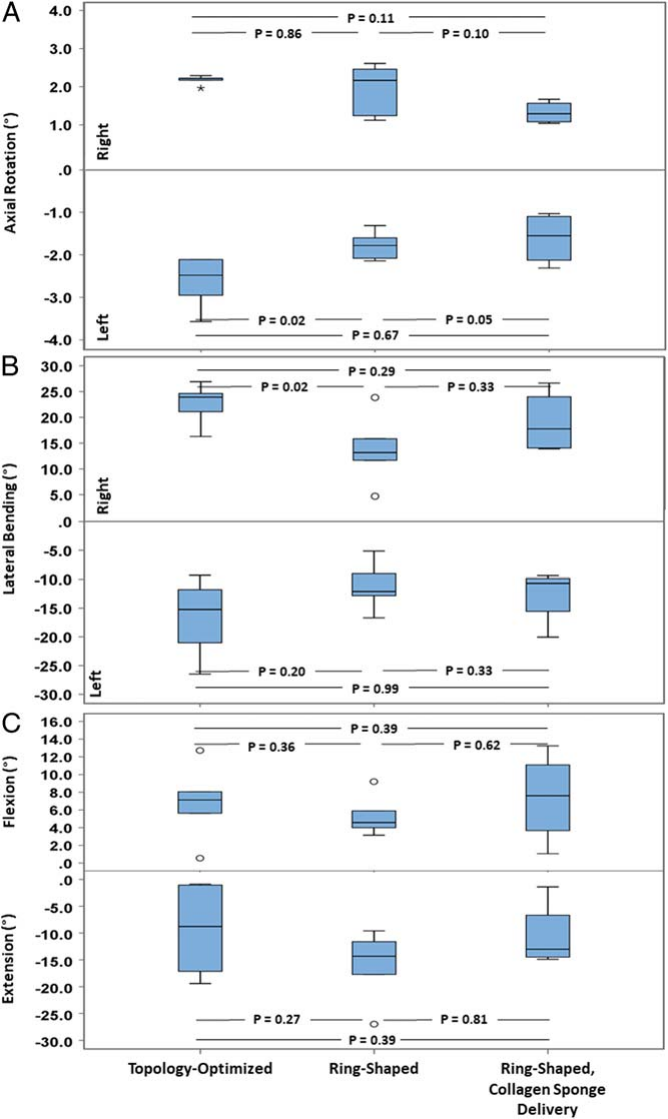
Box plots representing the distribution of range of motion for the complete cervical spine segment are presented. The median is shown by the thick horizontal line in each of the boxes. The bottom and top of the boxes depict the 25th and 75th percentiles, respectively. The whiskers represent the high and low values within 1.5 box lengths of the median. Outliers that fall between 1.5 and 3.0 box lengths of the median are plotted individually, as circles. Extreme outliers, which fall beyond 3.0 box lengths, are depicted as asterisks. Bending moments in each plane and direction are plotted separately: (A) left and right axial rotation, (B) left and right lateral bending, and (C) flexion and extension. *P* values for differences in medians between topology-optimized, ring-shaped, and ring-shaped with collagen sponge delivery are presented.

**Figure 4:**
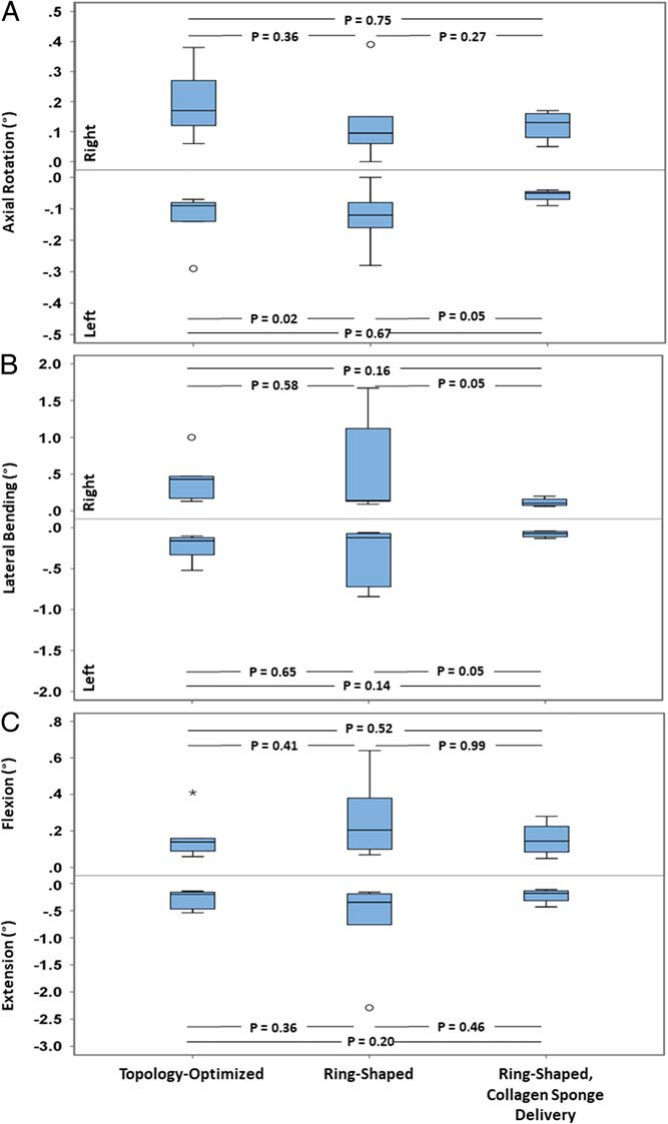
Box plots representing the distribution of range of motion for the treated functional spine unit are presented. The median is shown by the thick horizontal line in each of the boxes. The bottom and top of the boxes depict the 25th and 75th percentiles, respectively. The whiskers represent the high and low values within 1.5 box lengths of the median. Outliers that fall between 1.5 and 3.0 box lengths of the median are plotted individually, as circles. Extreme outliers, which fall beyond 3.0 box lengths, are depicted as asterisks. Bending moments in each plane and direction are plotted separately: (A) left and right axial rotation, (B) left and right lateral bending, and (C) flexion and extension. *P* values for differences in medians between topology-optimized, ring-shaped, and ring-shaped with collagen sponge delivery are presented.

Under axial torsion, median total segment rotations in all experimental groups were between 2.2° to the right and 2.5° to the left (Fig. 3). All of the FSU ROM under axial torsion were below 1.0°, with the median ROM ≤0.17° to the right and ≤0.12° to the left (Fig. 4).

In lateral bending, the total segment rotations in all experimental groups were much higher than they were for axial rotation or flexion/extension, with median ROM as high as 23.9° (Fig. 3). The median FSU ROM in lateral bending were below 1.0°, while the maximum ROM was 1.7° to the right and 0.8° to the left (Fig. 4).

The median total segment ROM in flexion was smaller than in extension with maximum flexion being 13.2° and 27.0° in extension, respectively (Fig. 3). The median FSU ROM in flexion and extension were small in both directions, with median ROM <1.0° (Fig. 4). Moreover, all motions were <0.6°, with the exception of one outlier, 2.3° in extension.

In summary, regardless of cage design or delivery method, FSU ROM were all small, with medians for all experimental groups <1° in all three planes. Although some statistically significant differences were found between groups, there were no clear trends. Moreover, given the small differences in magnitudes, these differences were likely not clinically significant.

For the control specimens, total cervical segment ROM was as high as 6.9° in flexion, and 25.1° in extension. In left and right lateral bending, motion ranged from 7.3 to 24.3°, and in left and right axial rotation, from 0.5 to 4.3°. For the FSU, ROM was as high as 1.1° in flexion and 2.8° in extension. In left and right lateral bending, motion ranged from 0.1 to 1.2°, and in left and right axial rotation, from 0.1 to 0.5°.

**Figure 5:**
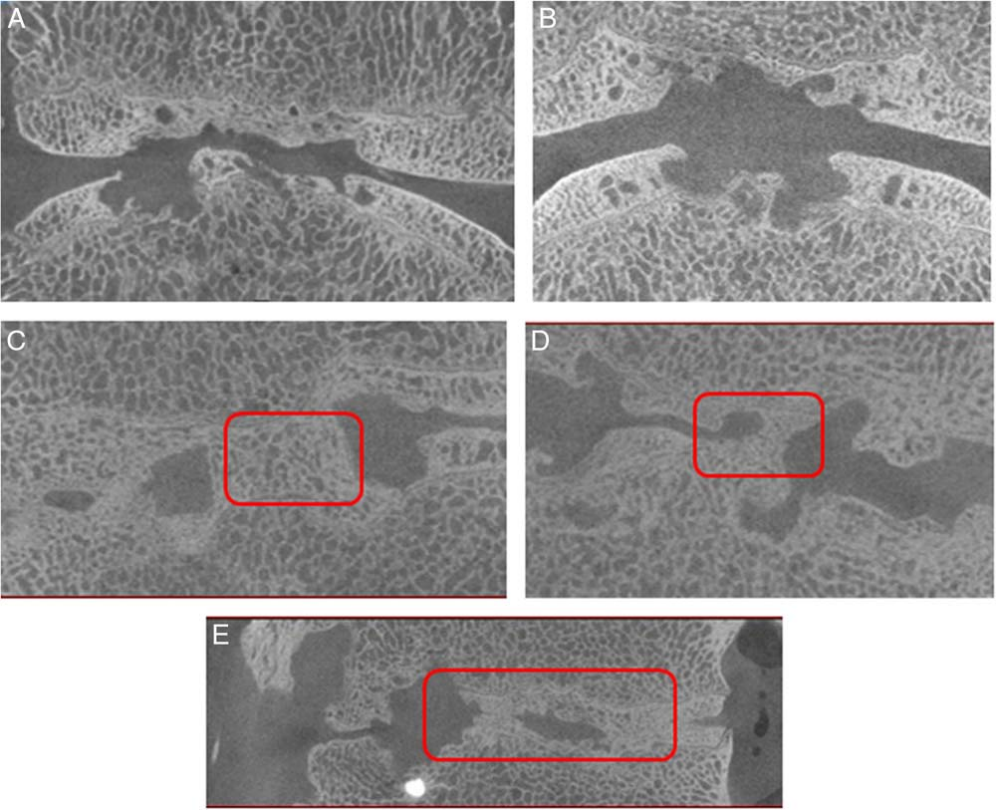
(A) Ring cage with no bone morphogenetic protein-2 (BMP-2) demonstrating no bony bridging on a selected coronal slice. (B) Topology-optimized cage specimen with no BMP-2 showing no bony bridging on a selected sagittal slice. (C) Ring cage specimen delivering 0.5 mg BMP-2 via adsorption showing complete bony bridging (in red outline). Empty space within bony bridge is occupied by poly-ε-caprolactone (PCL) cage. (D) Topology-optimized cage specimen delivering 0.5 mg BMP-2 via adsorption showing complete bony bridging (in red outline). Empty space within bony bridge is occupied by PCL cage. (E) Ring cage specimen delivering 0.5 mg BMP-2 via collagen sponge showing bony bridging (in red outline). Empty spaces next to bony bridging are location of PCL cage, which does not show up on nano-CT.

The evaluation of the nano-CT results indicated that the control specimens which had no BMP-2 did not achieve complete bony bridging across the fusion site (Table 1, Fig. 5). In contrast, all of the specimens with the ring designs using a collagen sponge for 0.5 mg BMP-2 delivery achieved complete bony bridging across the fusion site. In addition, 40% of ring designs with 0.5 mg adsorbed BMP-2 and 50% of topology-optimized cages with 0.5 mg adsorbed BMP-2 had complete bony bridging.

**TABLE 1:** Number of Experimental Specimens That Achieve Complete Bony Bridging as Assessed by Nano-CT After 6 Months *In Vivo*

Cage Design	BMP-2 Delivery Method	Total Number of Experimental Specimens	Number of Specimens With Complete Bony Bridging
Ring	0.5 mg BMP-2 adsorbed	5	2
Topology optimized	0.5 mg BMP-2 adsorbed	6	3
Ring	0.5 mg BMP-2 via collagen sponge	4	4

BMP-2 indicates bone morphogenetic protein-2; CT, computed tomography.

## DISCUSSION

A novel 3D printed, topology optimized, PCL device in a porcine model was studied following *in vivo* biologic incorporation by measuring the ROM in three planes as a function of topology optimization and BMP-2 delivery method. Overall, all experimental groups had limited ROM at the level of fusion while, the total cervical segments maintained some degree of flexibility in all three planes. These results indicate fusion had occurred at the treated level regardless of geometry and method of BMP-2 delivery.

In previous studies, thresholds of 2° of motion in flexion/extension and/or 1 mm of translation between the posterior elements have been used to indicate fusion.^51^–^56^ In the present study, all experimental specimens successfully restricted motion in flexion and extension to median values of less than 1.0°, indicating that fusion had occurred; however, the radiographic findings were not as consistent. A systematic review by Oshina *et al* 57 compared radiological fusion criteria for postoperative evaluation of cervical fusion. The authors concluded that the most commonly used fusion criteria, bridging trabecular bone between endplates and absence of radiolucent gaps between the graft and the endplate, were in fact subjective, even when CT imaging is used. After reviewing 10 fusion criteria in numerous articles, they concluded that clinical assessment of vertebral motion during flexion and extension was more reliable for evaluation of fusion than radiographic parameters.

The motion at the level of fusion was compared to previously reported healthy porcine spine biomechanical data at comparable cervical levels from Wilke *et al*.^49^ The ROM in the present study at the level of fusion were considerably lower than those reported by Wilke and colleagues for unfused, healthy spines. Specifically, in the present study, total FSU axial ROM was <1° compared to 2.5° in both directions in healthy, untreated, porcine spines. Similarly, in lateral bending, in the present study, ROM at the level of fusion was <1.9° compared to <17.0°, and <1.0° in flexion, compared to <10°, and <2.5° in extension compared to <6.0°,^49^ providing additional evidence that fusion had successfully occurred in the present study.

The majority of the previous studies of bioresorbable cages have been for PLA cages.^12^,^13^,^25^,^27^ Specifically, Yin and colleagues evaluated a 3D printed cervical cage composed of PLA-nanosized and β-tricalcium phosphate in an ovine model. Their biomechanical evaluation of the PLA cage, following *in vivo* incorporation, demonstrated decreased ROM in all three planes, compared to conventional, nonresorbable cages, indicating that a stiffer fusion construct may have been achieved.^58^ While these results were promising, generation of acidic products and inflammation, due to degradation of PLA, require longer follow-up to quantify.^12^,^13^,^27^

Previous studies have reported improved efficacy of BMP-2 when chemically adsorbed to PCL.^24^ Specifically, Patel *et al* 24 evaluated the bioactivity and bone growth of adsorbed BMP-2 via PCL scaffolding implanted subcutaneously *in vivo* demonstrated via subcutaneous implantation in mice that PCL scaffolding with adsorbed BMP-2-induced significantly more bone growth than other bonding methods. In the present study, there was no clinically significant difference in ROM between the experimental groups that compared delivery methods. However, complete bony bridging was observed in all of the specimens with the collagen sponge, as compared to roughly half of the specimens with adsorbed BMP-2, which may suggest that the collagen sponge is more effective than adsorption for solid devices.

### Cage Design

Two cage designs were studied: (1) a novel topology-optimized design with a rectangular shape and (2) a ring design, based on a commercially available device. Bony incorporation is a slow and gradual process. As such, cages play multiple roles, including providing resistance against external loads, buttressing of graft materials, and increasing neuroforaminal volume, until bony healing and fusion take over.^39^,^59^ The functional importance of the cage suggests the necessity for optimizing design. In the present study, all PCL cages implanted with BMP-2, regardless of design, were successful at restricting ROM at the level of the fusion, when compared to control specimens, implanted without BMP-2. However, in the present study, one major potential attribute of using PCL, that is, the resorption of the cage material, could not be investigated. Calculations based on the geometry of the cages suggest biological incorporation period of approximately four years would be necessary for the PCL cages to degrade completely, which was not possible in the present study.

### BMP-2

The results of the present study suggested that BMP-2 delivery via collagen sponge achieved more complete bony bridging than adsorption. This is likely due to a better retention and release profile as well as more efficient spatial distribution with the collagen sponge versus adsorption. Although it is difficult to place a standard collagen sponge in the interstices of a topology-optimized cage, recent work has demonstrated the ability to lyophilize a collagen sponge with mineral into 3D printed scaffolds with complex architecture resulting in increased bone formation in a porcine mandibular defect model.^60^ Therefore, future improvements in making hybrid 3D printed optimized cages with lyophilized or hydrogel delivery vehicles may enhance effective local BMP-2 delivery for spine fusion.

There were several limitations in the present study. First, due to the cost of a large animal study of this nature, the number of specimens was limited. Therefore, only four specimens were included as controls. Further, animals that suffered complications during surgery could not be replaced. In addition, the level of cervical fusion varied which may have influenced the findings.

## CONCLUSION

The results of the present study indicate that 3D printed bioresorbable PCL cages, regardless of design, or BMP-2 delivery method, successfully incorporated and restricted motion, resulting in fusion. Longer studies are needed to assess degradation.

**Key Points**

- The present study conducted a biomechanical evaluation of a 3D printed bioresorbable topology-optimized polycaprolactone (PCL) fusion cage in a large animal model, following 6 months *in vivo*.
- All PCL fusion cages, regardless of geometric design, successfully restricted motion to <1° at the level of fusion, which is below the clinically accepted threshold for fusion.
- Despite all experimental specimens achieving a biomechanically stable fusion, using CT, complete bony bridging was not observed in all specimens.
